# The pea aphid uses a version of the terminal system during oviparous, but not viviparous, development

**DOI:** 10.1186/2041-9139-4-10

**Published:** 2013-04-03

**Authors:** Ryan D Bickel, Hillary C Cleveland, Joanna Barkas, Caitlin C Jeschke, Amelie A Raz, David L Stern, Gregory K Davis

**Affiliations:** 1 School of Biological Sciences, University of Nebraska Lincoln, Lincoln, NE, 68588-0118, USA; 2 Department of Biology, Bryn Mawr College, Bryn Mawr, PA 19010, USA; 3 Janelia Farm Research Campus, Howard Hughes Medical Institute, Ashburn, VA, 20147, USA

**Keywords:** Development, Evolution, Pea aphid, Plasticity, Polyphenism, Terminal system

## Abstract

**Background:**

In most species of aphid, female nymphs develop into either sexual or asexual adults depending on the length of the photoperiod to which their mothers were exposed. The progeny of these sexual and asexual females, in turn, develop in dramatically different ways. The fertilized oocytes of sexual females begin embryogenesis after being deposited on leaves (oviparous development) while the oocytes of asexual females complete embryogenesis within the mother (viviparous development). Compared with oviparous development, viviparous development involves a smaller transient oocyte surrounded by fewer somatic epithelial cells and a smaller early embryo that comprises fewer cells. To investigate whether patterning mechanisms differ between the earliest stages of the oviparous and viviparous modes of pea aphid development, we examined the expression of pea aphid orthologs of genes known to specify embryonic termini in other insects.

**Results:**

Here we show that pea aphid oviparous ovaries express *torso-like* in somatic posterior follicle cells and activate ERK MAP kinase at the posterior of the oocyte. In addition to suggesting that some posterior features of the terminal system are evolutionarily conserved, our detection of activated ERK in the oocyte, rather than in the embryo, suggests that pea aphids may transduce the terminal signal using a mechanism distinct from the one used in *Drosophila*. In contrast with oviparous development, the pea aphid version of the terminal system does not appear to be used during viviparous development, since we did not detect expression of *torso-like* in the somatic epithelial cells that surround either the oocyte or the blastoderm embryo and we did not observe restricted activated ERK in the oocyte.

**Conclusions:**

We suggest that while oviparous oocytes and embryos may specify posterior fate through an aphid terminal system, viviparous oocytes and embryos employ a different mechanism, perhaps one that does not rely on an interaction between the oocyte and surrounding somatic cells. Together, these observations provide a striking example of a difference in the fundamental events of early development that is both environmentally induced and encoded by the same genome.

## Background

**Figure 1:**
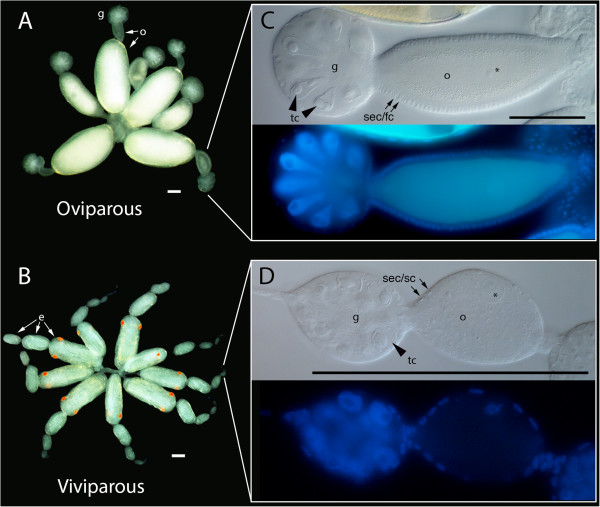
**Ovaries of sexual and asexual female pea aphids. A**. Oviparous ovary from a sexual female showing large germaria at the distal tip of each ovariole, each filled with one or two oocytes, including older ones that are yolk-filled. **B**. Viviparous ovary from an asexual female, showing embryos at various stages of development. Scale as A, but germaria and oocytes at the distal tips are almost too small to see. **C**. Bright-field close-up of oviparous germarium and previtellogenic oocyte. Note large nucleoli and teardrop shape of trophocytes as reported in [6] (arrowheads). Somatic epithelial or follicle cells form a cuboidal epithelium surrounding oocyte [10] (arrows). The oocyte (nucleus indicated by asterisk) will continue to increase in size during later vitellogenesis. Nuclei stained with DAPI are shown below, with the oocyte nucleus barely visible over autofluorescence. **D**. Bright-field and DAPI close-up of a viviparous germarium and previtellogenic diploid oocyte (nucleus indicated by asterisk), about 1/6th size of C, which will soon commence embryonic mitoses. Also in contrast to C, germarial trophocytes are spherical [6] (arrowhead) and the somatic epithelial or sheath cells surrounding the oocyte are squamous [63,64] (arrows). e, embryo; g, germarium; o, previtellogenic oocyte; sec/fc, somatic epithelial or follicle cells; sec/sc, somatic epithelial or sheath cells; tc, trophocytes. Scale bars are all 100 μm.

Aphids are small, hemimetabolous hemipteran insects that feed on the phloem sap of plants. All are cyclically parthenogenetic, meaning that they seasonally alternate between female parthenogenetic generations in which unfertilized diploid oocytes develop into females, and a sexual generation in which oocytes must be fertilized by sperm obtained from males. Asexual females, which occur typically in the spring and summer, maintain diploidy without sperm via a modified meiosis that, in effect, skips chromosomal reduction (reviewed in [1]). In all but the most basal groups of aphids, asexual females are also viviparous, their progeny completing embryogenesis within the ovaries prior to birth (Figure 1B). Sexual females are produced in the fall by asexual mothers that are exposed to shortened day lengths. Their ovaries contain oocytes (Figure 1A) that are fertilized at oviposition, initiating an embryogenesis that involves a frost-resistant diapause, allowing individuals to survive the winter and hatch in the spring [2,3].

During the evolution of aphids, the asexual-specific acquisition of viviparity was accompanied by significant changes in the oogenesis of asexual progeny [4]. For example, viviparous ‘oocytes’ start out smaller and grow less than oviparous oocytes [5-8]. In pea aphids, this means that viviparous oocytes range from one-third of the length of oviparous oocytes when first extruded to one-sixth just prior to vitellogenesis (Figure 1, compare arrows in C and D). The difference is probably due to the smaller size [7,9] and possibly reduced polyploidy [6] of viviparous as opposed to oviparous trophocytes (nurse cells), which support previtellogenic growth. Later, during vitellogenesis, oviparous oocytes continue to increase in size [6,10] while viviparous oocytes forego vitellogenesis to initiate mitotic divisions at a small size (Figure 1, compare A and B) [5-8]. Viviparous oocytes are also surrounded by fewer somatic epithelial cells than oviparous oocytes (Figure 1, compare C and D).

The differences between viviparous and oviparous oogenesis might have consequences for molecular patterning events. For example, the transient and truncated nature of viviparous oogenesis may preclude patterning mechanisms that rely on interactions between oocytes, somatic epithelial cells, and early embryos. We thus examined the expression of homologs of the *Drosophila* terminal patterning system in the pea aphid during oviparous and viviparous oogenesis and embryogenesis.

In *Drosophila,* the terminal system comprises a class of maternally expressed genes that specify the most anterior and posterior regions of the embryo: the labrum, cephalopharyngeal skeleton and portions of the optic lobes anteriorly, and structures posterior to abdominal segment A7 [11-14]. The system works through a receptor tyrosine kinase encoded by the gene *torso* (*tor*), the mRNA of which is transcribed in nurse cells and deposited into the oocyte [15]. After fertilization, *tor* mRNA is translated and the receptor is distributed uniformly over the surface of the early syncytial blastoderm [16] but activated only at the poles by a locally produced, diffusible ligand, whose movement in the perivitelline space is impeded by binding to its receptor [17,18]. Genetic evidence suggests that the ligand for Tor is the C-terminal fragment of the product of the terminal system gene *trunk* (*trk*), which is expressed in nurse cells and translated and secreted by the oocyte during oogenesis [19,20]. The cleavage of Trk requires the product of the terminal system gene *torso-like* (*tsl*) [20,21], which contains a membrane attack complex/perforin (MACPF) domain and is expressed and secreted by follicle cells located at the anterior and posterior poles [22-24]. Once secreted, Tsl is incorporated into the inner vitelline envelope at the poles [25], where it plays an as yet undefined role in the local processing of Trk, ultimately activating Tor at the poles of the early blastoderm and providing the spatial cue for the terminal system. Activated Tor, in turn, signals through the Ras-Raf-MEK-ERK/MAPK phosphorylation cascade [26], resulting in a gradient of activated ERK MAP kinase emanating from the poles just prior to cellularization [27]. In a concentration-dependent fashion, this kinase gradient then relieves transcriptional repression of the zygotic gap genes *tailless* and *huckebein*[28-31], allowing their expression at the anterior and posterior poles, where they are required to specify terminal fates [32,33].

Outside of dipterans, the most detailed report on the maternal terminal system is for the coleopteran *Tribolium.* In this holometabolous insect, as in *Drosophila*, both *trk* and *tor* mRNA are maternally provided to the oocyte, *tsl* is expressed in follicle cells lying anterior and posterior to the oocyte, and activated ERK MAP kinase is observed at the poles of the blastoderm [34-36]. RNA interference against *trk, tor*, or *tsl* causes a reduction in the serosa, an extraembryonic membrane derived from the anterior blastoderm, and posteriorly, loss of the entire abdomen [35,36]. Although at first glance these effects appear substantially different from those found in *Drosophila* terminal mutants, such differences are probably due to differences in the fate map of the blastoderm and the fact that *Tribolium* is a short germ insect wherein most of the posterior segments are generated after gastrulation from a posterior growth zone. Thus, the expression patterns and generalized function of the maternal terminal system, namely, specification of anterior and posterior regions of the blastoderm, appear to be well conserved in this insect. The genomes of the more basal holometabolous hymenopterans, however, thus far appear to lack *trk*[36,37], raising questions about whether the maternal terminal system operates in hymenopterans or in non-holometabolous insects, such as aphids.

Here we describe the aphid version of the terminal system by examining the ancestral oviparous mode of development. We also determine whether the system has been modified in the case of the derived viviparous mode. Our results suggest that while some aspects of the aphid maternal terminal system, for example, the expression of *tsl*, are conserved, other aspects, such as the timing and location of activated ERK, are derived. Our results also suggest that viviparous development in aphids does not use these components of the maternal terminal system, at least not in any way that resembles their use in *Drosophila* and *Tribolium*, raising questions about how asexual mothers are able to specify posterior fate in their daughter embryos.

## Methods

### Characterization of homologs in the pea aphid genome

To identify pea aphid terminal system genes we consulted both previous annotation efforts (for example, [38,39]) and PhylomeDB [40], as well as performed tblastx and tblastn searches of the pea aphid genome using verified terminal system orthologs. Phylogenetic analysis of torso-like genes was performed using a concatenation of three conserved amino acid blocks, one lying within the MACPF domain (GDFH…RFRE, LEEE…VFVY and YFSP…LLQL). Maximum-likelihood trees were constructed using the JTT substitution model in PhyML [41] implemented in Geneious Pro 5.6.4 [42]. The non-aphid insect Torso-like orthologs used in our phylogenetic analysis included: *D. pulex* (water flea, hxAUG25p1s4g245t1), *P. humanus corporis* (human body louse, XP_002423408), *A. mellifera* (honey bee, XP_394647), *Nasonia vitripennis* (jewel wasp, XP_001602735), *T. castaneum* (red flour beetle, EFA02884), *B. mori* (silkmoth, BGIBMGA009532), *D. plexippus* (monarch butterfly, EHJ73099), *D. melanogaster* (fruit fly, NP_524440), *A. gambiae* (mosquito, XP_307897). Signal peptide analysis of pea aphid torso-like genes was performed using SignalP 4.0 [43].

### Cloning of genes

Products of PCR were amplified from an *Acyrthosiphon pisum* (strain LSR1) mixed stage cDNA library and cloned into pGEM T-easy using the pGEM T-easy vector system kit (Promega, Madison, USA) or into pCR II-TOPO using the TOPO TA cloning kit (Life Technologies, Grand Island, USA). The following fragments were amplified using the indicated primer pairs: (i) three overlapping fragments of *tsl1*: 796 bp (5^′^-ACGAAUCUGCCGGAAAAACA-3^′^ and 5^′^-CTTCATGGAACCAAGCTCGT-3^′^), 532 bp (5^′^-AGCGATGGGCCTCGATGGGG-3^′^ and 5^′^-TGGCTCGTGAAGGTCGGTGC-3^′^), 604 bp (5^′^-TGGCAGGCGTTTACGGGCAG-3^′^ and 5^′^-AAGCCGGCGCCAAGGTTTTT-3^′^); (ii) two overlapping fragments of *tslr1:* 644 bp (5^′^-CGTTTTGTCCCGATTTGGACTGCC-3^′^ and 5^′^-CAGCCCGACCACCGACTGC-3^′^), an ≈1 kb fragment encompassing the entire coding region provided by E. Duncan and P. Dearden; (iii) a 866 bp fragment of *tor* that spans two intron-exon junctions (5^′^-AAGGGCACGCTGAAGACGGC-3^′^ and 5^′ ^CCGGTTGGCTGGGTTCGCAT-3^′^); (iv) a 705 bp fragment of *rl* (5^′^-GAATGGTCGTGTCGGCATTT-3^′^ and 5^′^-TGGTTTGAAGGGCAACGATT-3^′^); (v) a 744 bp fragment of *tll1* (5^′^-CCGGTCGACAAAACGCACCG-3^′^ and 5^′^-GCCGTCTGAGCGCCTCCTTG-3^′^).

### *In-situ* hybridization

Plasmids were cut with appropriate restriction enzymes and DIG-labeled sense and anti-sense RNA probes were transcribed with either T7 or SP6 polymerase using the DIG RNA labeling kit (SP6/T7) (Roche Diagnostics, Indianapolis, USA). *In-situ* hybridization was performed with material from stain LSR1 of *Acyrthosiphon pisum* using a modified version of previously described protocols [44,45]. Briefly, both oviparous and viviparous ovaries were dissected from mothers in PBS and then fixed in 4% formaldehyde in PBS for 30 min. Ovaries were then rinsed in PBS and taken through a MeOH series (50%, 70%, 90%, 100%) and stored at −20°C. Ovaries were rehydrated through a MeOH series (70%, 50%, 30% in PTw (PBS + 0.1% Tween-20)) and fixed in 4% formaldehyde in PBS for 20 to 30 min. Ovaries were then washed four times with PTw followed by a detergent solution (1.0% sodium dodecyl sulfate (SDS), 0.5% Tween-20, 50 mM Tris HCl (pH 7.5), 1.0 mM EDTA (pH 8.0), 150 mM NaCl) for 30 minutes to further permeabilize the tissue. Ovaries were then washed six times with PTw, prehybridized with a 1% SDS hybridization solution for 1 to 3 hours at 65°C, and then incubated with RNA probe at a concentration of ≈1.0 ng/μl in hybridization solution for 16 hours at 60 to 70°C, washed three times for 20 min each and four times for 30 min at 60 to 70°C with pre-heated hybridization solution, washed three times for 20 min each at room temperature with PTw, and blocked in PTw + 0.2% BSA for 1 hour before incubating at 4°C overnight with anti-DIG conjugated to alkaline phosphatase (Roche) at 1:2000. After 3 hours of washing with PTw, ovaries were washed three times for 5 min each in freshly prepared AP reaction buffer (5 mM MgCl, 100 mM NaCl, 100 mM Tris pH 9.5, 0.2% Tween-20) and then reacted in NBT/BCIP solution in the dark for 1 to 2 hours until the stain developed. Ovaries were then washed and counterstained with DAPI.

### Immunohistochemistry

Ovaries were dissected in 4% formaldehyde, fixed for 15 min, rinsed in PBS and stained according to [46] using monoclonal anti-ERK MAP kinase, activated (diphosphorylated ERK1&2) (M9692, Sigma-Aldrich, St. Louis, USA) [47] at a concentration of 1:200. Controls using the secondary antibody alone showed no staining.

### Real-time qRT-PCR

Sharpened tungsten needles were used to isolate germaria and oocytes (along with associated somatic epithelial cells) from approximately 20 oviparous and viviparous ovarioles in PBS. The tissue was placed in RNA*later* RNA stabilization reagent (Qiagen, Germantown, USA) and stored at 4°C. The RNA was purified using the SV Total RNA Isolation System (Promega, Madison, USA) and cDNA was made using the High Capacity cDNA Reverse Transcription Kit (Life Technologies, Grand Island, USA). Real-time qRT-PCR for pea aphid *tor* was performed on a Step One Plus real-time thermocycler (Life Technologies, Grand Island, USA) using Taqman Gene Expression Master Mix (Life Technologies, Grand Island, USA) and a PrimeTime qPCR Assay (Integrated DNA Technologies, Coralville, USA) that spanned an intron-exon junction (probe 5'-/56-FAM/CAG CAT CAA /ZEN/ATC GTG GTA CGC CAA C/3IABkFQ/-3' with primers 5'-ATT CGA CTC CCT TGC TAT TCG-3' and 5'-TGG GTG ACT TGC AGA CTT AC-3').

## Results

### The pea aphid genome contains homologs of some, but not all, members of the *Drosophila* maternal terminal patterning system

**Figure 2:**
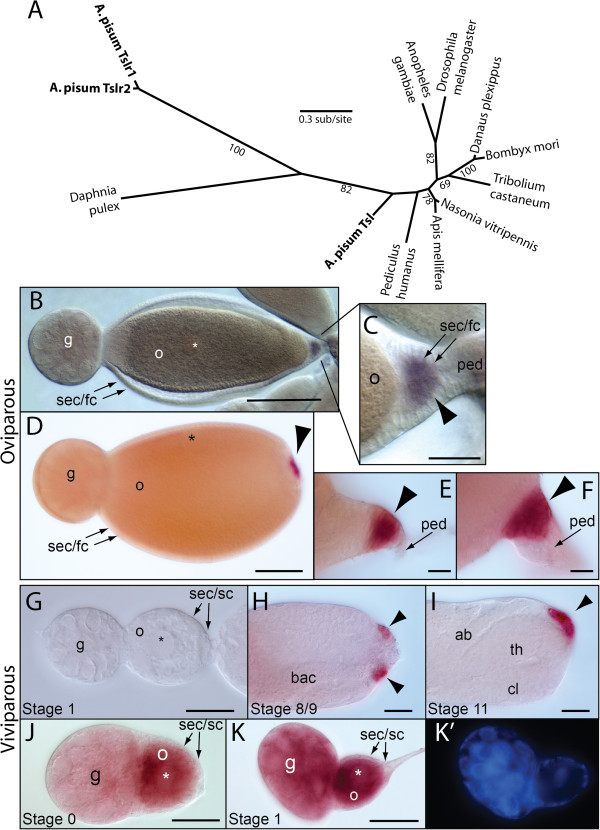
**Characterization and expression of pea aphid torso-like genes in oviparous and viviparous development. A**. Unrooted maximum-likelihood tree showing potential relationships between pea aphid (*A. pisum*) Tsl, Tslr1, and Tslr2 with several other arthropod torso-like homologs. Bootstrap percentages shown for all values >50%. **B-I**. *tsl* mRNA expression in oviparous (**B-F**) and viviparous (**G-I**) ovarioles. In oviparous ovarioles, *tsl* is expressed in a small group of somatic epithelial or follicle cells lying just posterior to late previtellogenic oocytes at the pedicel base (**B**). Arrowhead indicates domain in close-up (**C**). Observation of multiple focal planes showed that *tsl* is expressed in the follicle cells adjacent to the oocyte. The domain persists during vitellogenesis as oocyte becomes larger and cells undergo a series of morphological changes (**D-F**, arrowheads). The pedicel has been dissected away in D. *tsl* mRNA was not detected in any somatic epithelial or sheath cells surrounding viviparous oocytes (**G**, Stage 1). *tsl* is expressed in sheath cells only after embryo has begun to invaginate (Stage 8/9), first as a posterior circular domain at the base of the epithelial plug that is continuous with sheath cells surrounding the adjacent embryo (arrowheads in **H**, an optical cross section) and then as a non-circular domain following germ band invagination (**I**, arrowhead). In H,I the epithelial plug has been removed. **J,K**. *tslr1* mRNA is found in viviparous oocytes (**J**), probably because it is expressed in viviparous trophocytes in the germarium before being deposited into the oocyte (**K**). **K**^**′**^ shows nuclei stained with DAPI. ab, abdomen; cl, cephalic lobe; g, germarium; o, previtellogenic oocyte (nucleus marked with asterisk); ped, pedicel; sec/fc, somatic epithelial or follicle cells; sec/sc, somatic epithelial or sheath cells; th, thorax. Scale bars: 100 μm for **B** and **D**; 20 μm for **C**, **E-K**. Staging according to [8].

We searched for pea aphid homologs of members of the *Drosophila* maternal terminal system in the pea aphid genomic sequence [48], several of which had already been identified as the result of automated and manual annotation efforts [38] (Table 1). While the pea aphid genome possesses at least one copy of most of the terminal genes, it does not appear to possess homologs of either *fs*(*1*)*Nasrat* or *fs*(*1*)*polehole*, two related genes in *Drosophila* that are required for both terminal signaling and eggshell assembly [49,50]. Two pea aphid torso-like homologs, *torso-like* (*tsl*) and *torso-like related* (*tslr*), have been identified previously [38]. We have renamed *torso-like related* (*tslr*) as *torso-like related 1* (*tslr1*) in light of a third pea aphid torso-like homolog, which we have named *torso-like related 2* (*tslr2*). While phylogenetic analysis confirms that pea aphid *tsl* is a *torso-like* ortholog, *tslr1* and *tslr2*, themselves the product of a recent duplication, do not form a clade with pea aphid *tsl* (Figure 2A). The absence of *tslr* genes in other insects, however, suggests that the ancestral *tslr* is more likely the result of duplication within the aphid lineage and that the position of the *tslr* genes in our tree is due to sequence divergence of *tslr*.

**Table 1:** **Members of the *Drosophila *maternal terminal patterning system and their pea aphid homologs**

***Drosophila *gene**		**Pea aphid gene(s)**		**Accession number**
*fs*(*1*)*Nasrat*	*fs*(*1*)*N*	(absent)	–	–
*fs*(*1*)*polehole*	*fs*(*1*)*ph*	(absent)	–	–
*torso-like*	*tsl*	*torso-like*[38]	*tsl*	XP_001947286^a^
		*torso-like related 1* (aka *torso-like related;*[38])	*tslr1*	XP_001944406^a^
		*torso-like related 2*	*tslr2*	XP_003240865^a^
*trunk*	*trk*	(absent)		
*torso*	*tor*	*torso*[38]	*tor*	ACYPI009483-PA^b^
*rolled* (ERK MAP kinase)	*rl*	*rolled*[38]	*rl*	XP_001952106^a^

^a^XP accession numbers refer to Genbank; ^b^ACYPI accession numbers refer to AphidBase, official gene set 2.1 [39].

Although in *Drosophila* Trunk (Trk) appears to play an important role as the ligand of Torso [20], no *trk* homolog has been reported from the pea aphid genome [38] and indeed *trk* may be a derived feature of higher Holometabola, resulting from a duplication of the gene that codes for prothoracicotropic hormone (PTTH) [36]. We corroborated the absence of pea aphid *trk* by searching the pea aphid genome for all proteins containing both a signal peptide and a cystine knot, which is a stability motif found in several ligands, including Trk. Of these, none possessed significant sequence similarity to known Trk proteins. In spite of the absence of Trk, pea aphids do possess a single ortholog of *torso* (*tor*), the receptor tyrosine kinase activated by Trk and PTTH in *Drosophila*[20,51], as well as a single ortholog of *rolled* (*rl*), the gene that codes for ERK MAP kinase further downstream [38,52,53].

### Pea aphid *tsl* is expressed in posterior somatic follicle cells during oviparous but not viviparous oogenesis

Pea aphid *tsl* is expressed in a small group of posterior follicle cells that abut the oocyte during oviparous oogenesis at the end of previtellogenic growth (Figure 2B-C, arrowhead). The domain appears in follicle cells that lie between the oocyte and the base of the pedicel that connects to the lateral oviduct. During vitellogenesis the domain persists as the oocyte grows in size and the posterior follicle cells undergo a series of morphological changes (Figure 2D-F, arrowheads). In contrast, *in-situ* hybridization shows that *tsl* is not expressed in any of the somatic epithelial cells that surround the viviparous oocyte (Figure 2G) or the blastoderm embryo (data not shown). Only when the viviparous embryo begins to invaginate posteriorly is *tsl* expressed in a posterior subset of somatic epithelial cells, forming a circular domain that surrounds the base of the epithelial plug that connects to the adjacent, more mature embryo (Figure 2H, arrowheads). After the germ band has invaginated completely, this domain is no longer circular and is somewhat more focused (Figure 2I, arrowhead). Whether this later viviparous domain and the vitellogenic oviparous domain are related in either a developmental or evolutionarily sense is not clear.

Pea aphid *tslr1* is maternally expressed in the germ line during viviparous development, as we detected *tslr1* mRNA in the nurse cells and oocytes (Figure 2J-K). By *in-situ* hybridization, no such germ line expression of *tslr1* was observed in oviparous nurse cells or oocytes (data not shown). Although neither of our *tslr1 in-situ* probes was likely to be *tslr1*-specific, owing to the high level of sequence conservation between *tslr1* and *tslr2*, we presume that the observed signal in the germ line reflects *tslr1* transcripts, since we were unable to amplify *tslr2* from cDNA made from either oviparous or viviparous ovaries, despite repeated attempts using both conserved and *tslr2*-specific primers. In this regard, it is worth noting that, unlike pea aphid Tsl and Tslr1, the predicted protein Tslr2 (XP_003240865) lacks a signal peptide. The protein product is thus unlikely to be secreted if it is expressed at all and the gene may in general be nonfunctional.

### Pea aphid *tor* is expressed only at low levels in the germ line

We were unable to detect *tor* transcripts in the germ line of either oviparous or viviparous ovaries by *in-situ* hybridization, but we did detect low levels of transcript (28 <*C*_*t*_ < 36) from dissected germaria plus early oocytes by qRT-PCR. We were also able to clone a fragment of *tor* spanning two intron-exon junctions from cDNA derived from asexual ovaries, which included developing embryos. A possibility is that *tor* is expressed in the embryonic prothoracic gland, where it has recently been shown in *Drosophila* to act as the receptor for PTTH, which is required for proper regulation of ecdysteroid production and the closest paralog of Trk [36,51,54].

Despite the absence of *trk* in the pea aphid genome and inferred low levels of Tor at the surface the oocyte, we asked whether the *tsl* expression we observed during oviparous oogenesis might still provide a posteriorizing signal to either the developing oocyte or blastoderm embryo. To answer this question, we examined two possible downstream effects predicted by a functional terminal system: activation (phosphorylation) of ERK MAP kinase and transcriptional activation of *tailless.*

### Activated ERK MAP kinase is detected in nascent oocytes, distributed homogeneously in early oocytes, and later restricted to a posterior domain during oviparous but not viviparous oogenesis

**Figure 3:**
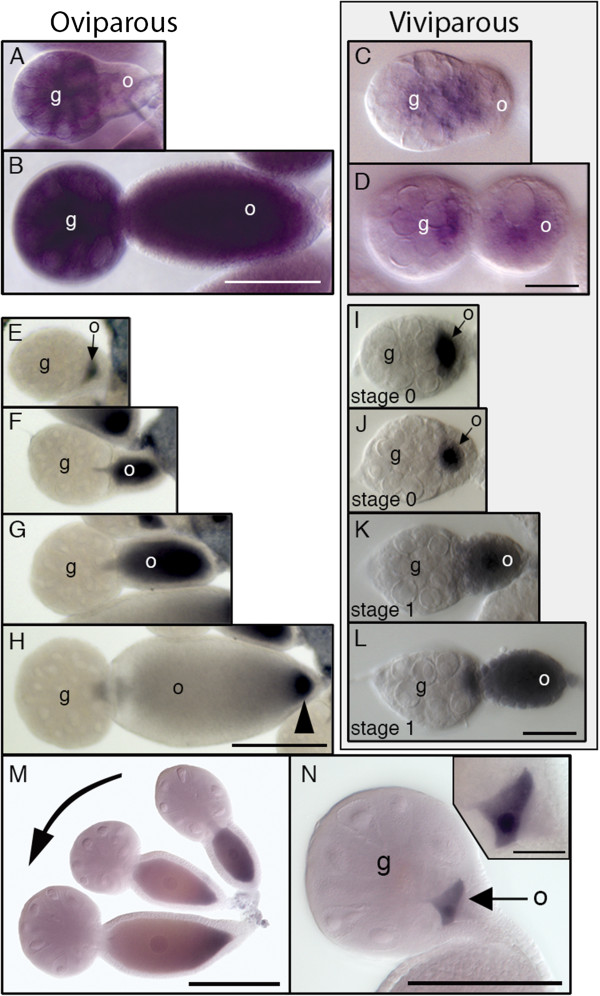
**Expression of the ERK MAP kinase gene *rolled *(*rl*) and distribution of activated ERK MAP kinase during oviparous and viviparous oogenesis. A-D**. *rl* mRNA is detected in the trophocytes of the germaria and in young oocytes during both oviparous (**A,B**) and viviparous (**C,D**) oogenesis. **E-M**. Activated ERK MAP kinase (dpERK) is first found in early oocytes prior to being extruded from germarium during both oviparous (**E** and **M**, see inset for close-up) and viviparous (**I,J**) development. Following extrusion, dpERK is distributed throughout the oocyte in oviparous (**F,G**) and viviparous (**K,L**) cases. During late previtellogenesis of oviparous oocytes, however, dpERK becomes restricted to the most posterior region of the oocyte (**H**, arrowhead). The progressive nature of this restriction occurs as the oocyte increases in size (**M**, arrow indicates younger to older oocytes). g, germarium; o, oocyte. Scale bars: 100 μm for **A,B**; 20 μm for **C,D**; 100 μm in **E-H;** 20 μm for **I-L;** 100 μm in **M,N** (20 μm in inset). Staging according to [8].

First, we performed *in-situ* hybridization to *rl*, which codes for ERK MAP kinase [53]. As in *Drosophila*[52], the gene is maternally expressed in the pea aphid germ line (Figure 3A-D). We then visualized the activated form of ERK MAP kinase using an antibody that is specific to diphosphorylated ERK (dpERK) [47,55]. We first detected dpERK within a single cell of the germaria of both oviparous and viviparous ovarioles, in the most posterior oocyte that is about to be extruded (Figure 3E,I,N). In these oocytes, dpERK is found throughout the cytoplasm and nucleus, with potentially higher levels in the nucleus (for example, see insert in N). This distribution persists during extrusion and early previtellogenic growth (Figure 3E-G,I-L). In the viviparous case, dpERK disappears quickly, subsiding in a homogeneous fashion, just prior to the first mitotic division of embryogenesis, during which we did not observe dpERK (data not shown).

In the oviparous case, dpERK does not subside in a homogeneous fashion; we instead observed that dpERK is cleared progressively from most of the oocyte starting at the anterior, but is retained in a posterior domain near the previously described *tsl*-expressing follicle cells (Figure 3H, arrowhead). As the oocyte grows, the domain occupies relatively less space in the oocyte but does not itself shrink in size (Figure 3M). The stage at which we observe posteriorly restricted dpERK is equivalent to the stage at which we observe the onset of *tsl* expression in posterior follicle cells (compare Figure 3H to Figure 2B-C). The domain subsequently disappears during vitellogenesis and we did not observe dpERK in newly laid oviparous blastoderm embryos (data not shown). Importantly, we did not observe progressive clearing of dpERK or the retention of a posterior dpERK domain during viviparous oogenesis.

### Pea aphid *tailless* is not expressed in a posterior domain during either oviparous or viviparous embryogenesis

**Figure 4:**
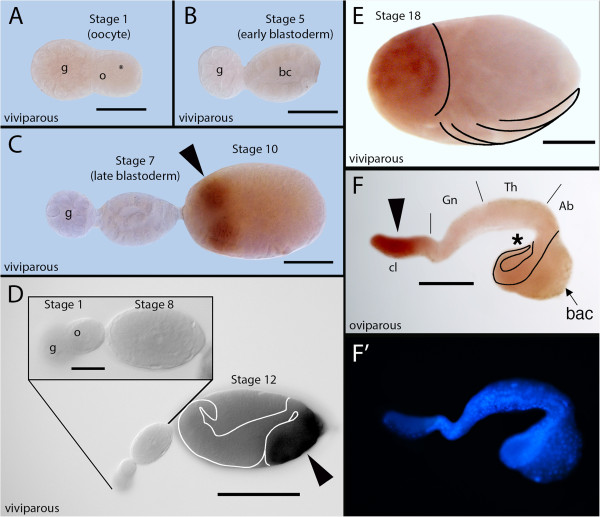
**Expression of *tailless *(*tll*) during viviparous and oviparous embryogenesis*. *A-E**. *tll* expression in viviparous embryos*. tll* mRNA is not detected in germaria or oocytes (stage 1 in **A**), early or late blastoderm embryos (stage 5 in **B**, stage 7 in **C**), or prior to invagination (stage 8 in close-up in **D**). *tll* mRNA is first detected after germ band formation in two anterior bilateral spots within cephalic lobes, corresponding to developing brain (stage 10 in **C**, arrowhead). Domains spread to full extent of cephalic lobes (stage 12 in **D**, arrowhead) and persist but become more nuanced, reflecting development of eye and central nervous system (stage 18 in **E**, antennae and limbs indicated by arrows). In all panels, anterior to the left; **E** dorsal, up. Stage 10 in **C**, ventral view. Stage 12 in **D**, lateral view of embryo with anatrepsis, causing inversion of embryonic dorsal-ventral and anterior-posterior axes within egg. Cephalic head lobes (staining) not inverted but displaced towards posterior (right) of egg. Embryo is outlined by white lines. **E**, lateral view following katatrepsis, returning head lobes to anterior of egg. Extent of head lobe and outlines of leg appendages indicated by black lines. **F**. Lateral view of *tll* expression in head lobes (arrowhead) of oviparous embryo still forming abdominal segments (~6 days after egg laying). Note absence of staining (asterisk) at posterior abdomen, which curls under embryo ventrally (bacteriome located dorsally, arrow). Posterior abdomen outlined by black lines; approximate extents of cephalic lobe, gnathos, thorax, and abdomen indicated by vertical black lines. Nuclei stained with DAPI shown in **F**^**′**^. ab, abdomen; bac, bacteriome; bc, blastocoel; cl, cephalic lobe; g, germarium; gn, gnathos; o, oocyte; th, thorax. Scale bars: **A-C**, 20 μm; **D**, 100 μm (inset, 20 μm); **E**, **F**, 100 μm. Staging according to [8].

Of the two well-known gap gene targets of the *Drosophila* terminal system, the pea aphid genome does not possess *huckebein*, but does possess a single ortholog of *tailless* (*tll*) (accession number XP_001945915) [38]. Based on descriptions of *tll* expression in other insects, we expected that *tll* would be expressed in the posterior blastoderm and the anterior head lobes at the germ band stage. We observed that *tll* is expressed in the anterior head lobes in both viviparous and oviparous germ bands. In viviparous embryos, this pattern appears first in the germ band stage as two dorsolateral spots (Figure 4C, arrowhead) and develops throughout anatrepsis and katatrepsis (Figure 4D-E, arrowhead in D). Although we observed a similar anterior domain in the head lobes of oviparous embryos (Figure 4F, arrowhead), we did not observe expression in the posterior during early embryogenesis. A similar cephalic pattern is reported in *Drosophila*, *Tribolium*, *Nasonia,* and the honey bee, the activation of which is likely to be independent of the terminal system [34,56-58].

## Discussion

Previous studies of differential gene expression between aphid oviparous and viviparous development have taken a largely transcriptomic approach to identify differentially expressed genes (for example, [59]). We have instead taken a candidate gene approach, investigating the expression of terminal system genes in the pea aphid to identify differences between oviparous and viviparous oogenesis. Here we report two: a difference in the expression patterns of *torso-like* (*tsl*) and a difference in the distributions of activated ERK MAP kinase (dpERK) in the oocyte. We also report aspects of aphid development that are shared between the oviparous and viviparous modes but differ from more derived holometabolous insects, namely the presence of dpERK in the oocyte, the lack of an anterior domain of *torso-like* (*tsl*), and the lack of a posterior domain of *tailless* (*tll*). Together, these observations suggest the following: (i) dpERK plays a role in oocyte differentiation in aphids, (ii) an aphid version of the terminal system operates at the posterior of oviparous but not viviparous oocytes, possibly via a transduction mechanism other than Trk-Tor, and (iii) the roles played by *tsl* and *tll* in specifying terminal fates have evolved within insects.

### dpERK may play a role in the differentiation of aphid oocytes

We detected dpERK throughout the cytoplasm and nucleus of early oviparous and viviparous oocytes, but not in nurse cells. Although dpERK is not found in *Drosophila* oocytes [60], it is found in previtellogenic oocytes of *Tribolium*, first in the nucleus and possibly later in the cytoplasm (see Figure 1J-K in [61]), and thus may be found in the oocytes of other insects. In the pea aphid, dpERK appears first in nascent oocytes, prior to extrusion from the germarium, suggesting that it may play a role in oocyte differentiation. In this respect, it is important to note that within oviparous and viviparous germaria, we observe dpERK only in the most posterior oocyte (for example, Figure 3N), the one next in line to be extruded. This is despite the fact that, in both oviparous and viviparous germaria, multiple presumptive oocytes simultaneously exhibit morphological differentiation prior to extrusion [6,9,62]. It would thus appear that the activation of ERK MAP kinase represents a relatively late step in the process of oocyte differentiation that takes place within the germarium.

### An aphid ‘terminal’ system may operate at the posterior of oviparous but not viviparous oocytes

**Figure 5:**
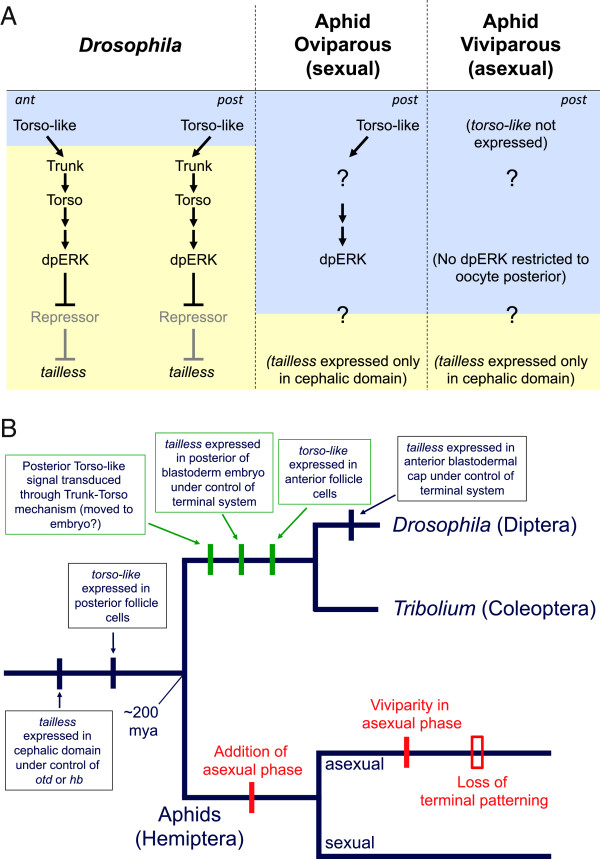
**Summary of results and evolution of terminal system and expression of *tailless*. A**. Comparison of terminal system architecture between *Drosophila* and the pea aphid in its oviparous mode. Gray and yellow shading indicate events occurring during oogenesis and embryogenesis, respectively, with border representing fertilization. Note that the aphid terminal system only operates in the posterior and that the signal transduction mechanism of Torso-like activity and the targets of dpERK remain unknown. Also note that these aspects of the aphid terminal system do not operate during viviparous oogenesis. **B**. Proposed steps in the evolution of the terminal system in *Drosophila* and aphids, with asexual and sexual developmental programs of aphids shown as separate evolutionary trajectories. Although steps shown in green are depicted as gains in the lineage leading to *Drosophila* and *Tribolium*, they could just as parsimoniously be represented as losses in the lineage leading to aphids. The order of steps between nodes is arbitrary with the exception of the acquisition of viviparity in asexual aphids, which we suggest either pre-dated or coincided with the loss of terminal patterning. Neither A nor B makes reference to expression of aphid *tslr1* or early homogeneous dpERK in the oocyte.

In both *Drosophila* and *Tribolium*, *tsl* is expressed in groups of somatic follicle cells lying just anterior and posterior to the oocyte [22,23,35]. In the pea aphid, in contrast, we find that *tsl* is expressed only in a posterior domain, abutting the oocyte only in the oviparous case (Figures 2 and 5A). The anterior domain may have been acquired in the lineage leading to *Drosophila* and *Tribolium* (Figure 5B) or lost in the lineage leading to aphids. Our observations that *tsl* is not expressed in any of the somatic epithelial cells that surround the viviparous oocyte, and that dpERK is not restricted to the posterior of the viviparous oocyte, suggest that aphids dispense with their version of the terminal system during viviparous development. This change in molecular patterning may have been ushered in by the asexual-specific acquisition of viviparity (Figure 5B), which was accompanied by multiple changes in cell function and morphology.

One set of viviparity-associated changes that could explain the loss of *tsl* expression concerns the somatic epithelial cells that surround the extruded oocyte, referred to as follicle cells in the oviparous context and sheath cells in the viviparous context [63,64]. Although generally considered homologous (for example, [8]), follicle cells and sheath cells differ in both morphology and function, in order to play different roles during oviparous and viviparous development, respectively [10,63-65]. These differences must arise because these two cell types differentiate differently and it may be that the presumably derived process of sheath cell differentiation simply precludes *tsl* expression during viviparous oogenesis.

A viviparity-associated change that may explain why we do not observe a progressive clearing of homogeneous dpERK in viviparous oocytes is the fact that viviparous oogenesis is truncated, limited to a shortened previtellogenic growth phase. There may simply not be enough time to clear dpERK prior to the first mitotic division.

A final consideration is scale. The smaller size of the viviparous oocyte combined with a low density of sheath cells may preclude the use of an external signaling mechanism, such as Tsl, as such a mechanism may not be able to deliver an adequately focused signal.

If oviparous oocytes specify posterior fate through an aphid terminal system involving *tsl* and dpERK, then presumably viviparous oocytes employ a different mechanism, perhaps one that does not involve interactions between the oocyte and surrounding somatic cells. One possibility is that viviparous oocytes and embryos rely more heavily on the aphid version of the insect posterior system. In *Drosophila*, posteriorly restricted Nanos protein is capable of downregulating a transcription factor known to be required for proper repression of *tll*, possibly downstream of dpERK [66]. Like *Drosophila*, Nanos protein is found at the posterior of pea aphid viviparous oocytes and embryos [67]. Although we do not observe posterior derepression of *tll* in aphids, it is conceivable that Nanos acts as a viviparous understudy, fulfilling the roles Tsl and dpERK play during oviparous development. Such an internal mechanism may be able to provide the more focused activity required by a relatively small viviparous oocyte, as compared with an external signal that may only be able to break the symmetry of a larger target.

### The aphid terminal system may use an alternate means of signal transduction

In *Drosophila*, dpERK is not detected at the poles of the blastoderm embryo until after 12 nuclear divisions [27]. This is presumably because Torso (Tor) protein is not detected until the ninth nuclear division [16], as maternally provided *tor* mRNA is not translated until after fertilization. Our results in the pea aphid suggest that *tor* is expressed at low levels, if at all, in the germ line. It is thus possible that ERK activation in the oocyte may be due to a *tor*-independent mechanism. In *Drosophila*, ERK is activated during embryogenesis via several receptor tyrosine kinases other than Tor, including epidermal growth factor receptor (EGFR), Heartless and Breathless [68]. G-protein-coupled receptors have also been reported to work through the ERK MAP kinase pathway [69]. Additionally, given that PTTH has been shown to activate Tor in the *Drosophila* prothoracic gland [51], and that this activity requires Tsl [36], it is worth noting that a pea aphid homolog of *PTTH* has been identified and is expressed in extended germ band embryos and at low levels in the oviparous germ line [70]. Whether any of these receptors or ligands is required for the ERK activation we observe in the pea aphid oocyte remains to be determined.

The fact that during oviparous oogenesis the onset of *tsl* expression in posterior somatic follicle cells coincides with the retention of a posterior domain of dpERK in the oocyte suggests that, as in *Drosophila* and *Tribolium*, secreted Tsl plays a role in activating dpERK. While this proposal awaits testing by functional disruption of *tsl*, if *tsl* is indeed required for the posterior domain of dpERK, the lack of *trunk* (*trk*) in the pea aphid genome, combined with the possible lack of *tor* expression in the germ line, suggests a mechanism of signal transduction that relies on a ligand other than Trk and possibly on a receptor other than Tor; one that is present in the oocyte membrane capable of transducing a signal to the oocyte, rather than to the embryo as in *Drosophila* (Figure 5A). In addition, because posterior dpERK does not apparently persist into embryogenesis, any patterning of the embryo by dpERK must necessarily be indirect, passed along to embryonic nuclei via an as yet unidentified cytoplasmic messenger.

### The role of tailless in patterning the brain is ancestral for insects, but its posterior function may be derived

In *Drosophila*, the gap gene *tailless* (*tll*) is expressed in cap-like domains at the anterior and posterior termini of the blastoderm embryo in response to signaling from the terminal system [32,56]. The mechanism of activation appears to be relief of repression, with Groucho as the likely repressor [28,29]. The anterior cap domain subsequently resolves into a dorsolateral stripe (cephalic domain) that is largely independent of the terminal system and is required for embryonic brain development [56,71]. With some variation, this pattern is largely conserved among dipterans (for example, [72,73]). Outside of Diptera, in *Tribolium*, the cephalic domain of *tll* is present in the germ band, but the earlier anterior blastodermal cap observed in *Drosophila* is absent [34]. This is despite the fact that *tsl* is expressed in the anterior follicle cells and there is an anterior blastodermal domain of dpERK, suggesting that *tll* came under the control of the anterior terminal system in the dipteran lineage (Figure 5B). Posteriorly, the posterior cap domain is present and appears to be regulated by the terminal system, as evidenced by the loss of this domain in *tsl* and *tor* RNAi embryos [34,35]. Both the cephalic and the posterior domains of *tll* are also found in hymenopterans, although the means of restricting *tll* activity to the posterior appears to differ: activation by *orthodenticle* in the case of *Nasonia* and localization of maternally provided *tll* mRNA in the case of the honey bee [57,58].

In the pea aphid, we find that *tll* is expressed in the anterior cephalic domain but not in the posterior blastoderm embryo (Figures 4 and 5A), and thus presumably is not under the control of the terminal system. This confirms previous suggestions, based on comparisons with the vertebrate homolog of *tll*, *Tlx*, that the ancestral role of *tll* is in the embryonic brain [74,75]. Whether posterior *tll* expression was gained in the lineage leading to holometabolous insects (Figure 5B) or lost in the lineage leading to aphids is not yet clear.

## Conclusions

The data presented here suggest that activated ERK MAP kinase plays a role in oocyte differentiation in the pea aphid. The data also suggest that aspects of the *Drosophila* terminal system are conserved in the pea aphid during oviparous development, but are dispensed with during viviparous development. Future investigation should reveal whether other early fundamental events also differ between oviparous and viviparous development and shed light on how such different processes are encoded by a single genome.

## Abbreviations

Ab: Abdomen; AP: Alkaline phosphatase; Bac: Bacteriome; Bc: Blastocoel; BSA: Bovine serum albumin; Cl: Cephalic lobe; DAPI: 42,6-diamidino-2-phenylindole; DIG: Digoxigenin; dpERK MAP kinase: Diphosphorylated (activated) extracellular signal-regulated mitogen activated protein kinase; EGFR: Epidermal growth factor receptor; ERK MAP kinase: Extracellular signal-regulated mitogen activated protein kinase; G: Germarium; Gn: Gnathos; MACPF: Membrane attack complex/perforin; NBT/BCIP: Nitroblue tetrazolium/5-bromo-4-chloro-3-indolyl phosphate; O: Oocyte; PBS: Phosphate-buffered saline; PCR: Polymerase chain reaction; Ped: Pedicel; PTTH: Prothoracicotropic hormone; PTw: PBS + 0.1% Tween-20; qRT-PCR: Quantitative reverse transcription polymerase chain reaction; rl: Rolled; SDS: Sodium dodecyl sulfate; sec/fc: Somatic epithelial or follicle cell; sec/sc: Somatic epithelial or sheath cell; tc: Trophocyte/nurse cell; th: Thorax; tll: Tailless; tor: Torso; trk: Trunk; tsl: Torso-like; tslr: Torso-like related.

## Competing interests

The authors declare that they have no competing interests.

## Authors’ contributions

RDB, GKD, and DLS designed the experiments. RDB, GKD, HC, JB, CJ, and AZ performed the *in-situ* hybridizations, immunostainings, and qPCR. GKD, RDB, and DLS drafted and revised the manuscript. All authors read and approved the final manuscript.
